# Bow-Legs

**Published:** 1850-03

**Authors:** 


"*Bow-Legs.*" By E. F. Lonsdale, Esq., Assistant Surgeon to the Orthopædic Hospital.

This affection, outward curvature of the tibia and fibula, does not necessarily arise from any constitutional taint; it may originate simply from the bones being too weak to support the weight of the body. If there be general debility, or if the child has been badly nourished, and exposed to impure air, attention to these circumstances should of course be our first object. When, however, the case has not originated in these conditions, or under any circumstances, if the affection is severe, we should use mechanical means of relief. In cases of bow-legs, Mr. Lonsdale observes, though some children do certainly "grow out" of the deformity, the great majority will get worse, if left unchecked. As to the mechanical means to be adopted, Mr. Lonsdale says:

For the common slight form of curvature, all that is required is, two calico bandages, two small wooden splints, and six webbing straps with buckles—three for each leg, two broad ones, and one narrow. The splints should be long enough to extend from the condyle of the femur down to the edge of the sole of the foot; they are to be padded at each end with a pad large and thick enough to cover the inside of the knee and the inner malleolus. If the pad be made in one piece, the two ends should be thicker than the centre. The splints should be quite as wide as the thickness of the leg—if anything a little wider; the object of this being to remove the pressure from the fore and back part of the limb, and making the straps press upon the outer side principally. They are applied as follows:—Bandage the leg evenly, from the toes upwards to a little above the knee; the use of this being to equalize the circulation, and guard the skin from the irritation and friction of the straps and pads. The splint is then to be placed on the inside of the leg, so that the upper pad may rest on the inside of the knee and the lower one on the inner ankle; care should be taken not to allow the splint to fall forwards, or otherwise the lower end of it will press against the instep, and interfere with the child's walking, independently of preventing the pressure acting in the most favorable manner on the curve of the bone; the lower pad should also be thick enough to throw the end of the splint away from the foot. The knee and ankle straps should be attached to the splint in a position to correspond to these two joints when fastened, while the centre one should be left moveable to allow of its position being altered at pleasure, to accommodate itself to the most curved part of the bone, opposite to which it should be placed. When the straps are tightened (and they should only be slightly so at first), the centre one should make the most pressure; care being taken to avoid pain and irritation on the skin, as well as swelling of the foot, which will occur if the ankle strap be too tight. If the inner malleolus be very prominent, and the skin very liable to become irritated by slight pressure, the lower end of the splint may be hollowed out opposite to the ankle; as a general rule, however, if the pad be made soft and thick, this is not required. The child should wear shoes, and not boots; the latter giving no real support to the ankle joint, and only cause irritation to the skin when the straps are tightened on the pad that lies upon the boot: the ormer leaves the lower part of the leg quite free, and allows the ankle strap to be adapted to the lower end of the curve with more precision and effect than it would be if made to tell upon the hard leather of the boot.

The rules to be observed during the time the splints are being worn are few and simple: to keep up sufficient pressure on the bone by gradually tightening the straps, more particularly the one or the two (if more than one are employed) that act upon the centre or most curved part of the bone. Never let the child stand without splints; when it is necessary to take them off to wash the child, let it lie down until they are reapplied. To guard against irritation of the skin by relieving the pressure, if the child complains of pain. To attend to the general health of the child by giving strengthening and other medicines if required, and procuring change of air, if practicable. Not to leave the splints off at once when the bones have become sufficiently straightened; to do so by degrees, beginning with an hour or two a day, and increasing the time every second or third day; the object of this is to avoid throwing the weight of the body suddenly on the ankle and knee joints, by doing which an equal strain might be made upon them, and a weakness produced, and a tendency to reoccasion the curvature, owing to the bones not being kept in the perpendicular position. There is one other point not mentioned, with regard to the thickness of the pad at the knees; if there be any tendency to knock-knees, they should be made very thin, otherwise they only prevent the approximation of the thigh bones, and throw the feet outwards, and bring the strain upon the knee-joints.

The length of time the splints will require to be worn must depend upon the period during which the deformity has existed, its degree of severity, and the age of the patient; as a general rule, the time will vary from six to twelve months; of course this being influenced by the above circumstances, as to whether it should be longer or shorter*.—*From Braithwaite's Retrospect.*

*In these cases, a useful medicine is the phosphate of lime, ten or fifteen grains once or twice a day, mixed with the food of the child.—Ed.

